# Additive effects of GLY-200 (oral pharmacologic duodenal exclusion therapy) and GLP-1R agonist in obesity management

**DOI:** 10.1016/j.molmet.2025.102287

**Published:** 2025-11-19

**Authors:** Taylor L. Carlson, Mark Fineman, Stace Kernodle, Chelsea R. Hutch, Christine Bryant, Kevin Colbert, Randy J. Seeley, Ashish Nimgaonkar

**Affiliations:** 1 Glyscend Therapeutics, Inc., Baltimore, MD, USA; 2 Department of Surgery, University of Michigan, Ann Arbor, MI, USA; 3 Center for Bioengineering Innovation and Design, Department of Biomedical Engineering, The Johns Hopkins Hospital, Baltimore, MD, USA; 4 Division of Gastroenterology, Department of Medicine, The Johns Hopkins Hospital, Baltimore, MD, USA

**Keywords:** GLY-200, Duodenal exclusion, Bariatric surgery, GLP-1RA, Combination therapy, Weight loss, Weight maintenance

## Abstract

Type 2 diabetes and obesity impact billions of people and the global prevalence is only growing. Current treatment options, which include pharmacotherapy, e.g., GLP-1 receptor agonists (GLP-1RA) and bariatric surgical approaches have limitations. GLY-200 is an investigational clinical-stage oral non-absorbed polymeric drug designed to target proximal intestinal mucin and enhance its barrier function, emulating duodenal exclusion physiology for the treatment of diabetes and obesity. The efficacy of GLY-200 as a monotherapy and in combination with semaglutide, a leading GLP-1 receptor agonist (GLP-1RA) for obesity weight management was evaluated in diet-induced obesity (DIO) mice. Significant improvements in metabolic parameters were seen in mice treated with GLY-200 monotherapy. Moreover, an additive effect was observed when GLY-200 was combined with semaglutide, resulting in enhanced weight loss and metabolic improvements beyond those achieved with either treatment alone. GLY-200 showed promise as a weight maintenance drug, significantly blunting the weight rebound seen after GLP-1RA discontinuation. Phase 2a data from patients with type 2 diabetes (T2D) showed reductions in fasting and postprandial blood glucose, improved fasting lipid profiles, and progressive weight loss with GLY-200 treatment. These findings suggest that GLY-200, in combination with GLP-1RAs, holds promise as a novel therapeutic strategy for obesity, potentially offering a valuable approach for GLP-1RA dose reduction or weight maintenance following GLP-1RA discontinuation.

## Highlights

• GLY-200 mimics duodenal exclusion, improving metabolic parameters in diet induced obese mice.
• Combination of GLY-200 and semaglutide yields additive weight loss and glycemic control.
• GLY-200 supports weight maintenance after GLP-1RA discontinuation in preclinical models.
• Oral duodenal exclusion offers a novel adjunct to GLP-1RAs.

## 1 Introduction

The global prevalence of obesity and its associated metabolic disorders, such as type 2 diabetes (T2D) and cardiovascular complications, continues to rise, demanding innovative therapeutic approaches. GLP-1 receptor agonists (GLP-1RAs) have become significant players in T2D and obesity management. These drugs mimic the effects of glucagon-like peptide-1 (GLP-1), an incretin hormone released in response to nutrient ingestion. GLP-1 is crucial in glucose homeostasis, appetite regulation, and gastric emptying. GLP-1RAs bind to GLP-1 receptors in the brain and pancreas, leading to increased glucose-dependent insulin secretion, suppressed glucagon secretion, delayed gastric emptying, and reduced appetite [1]. These effects contribute to weight loss and improved glycemic control in patients with obesity and T2D. Numerous clinical trials have documented significant weight loss and improvements in HbA1c levels with various GLP-1RAs [1,2].

Despite these benefits, the currently available GLP-1RAs have limitations such as cost, injectable administration, side effects, and limited long-term efficacy. Additionally, not all patients respond to these drugs in part due to the complexity of obesity, stemming from the interplay of genetic, environmental, and hormonal factors. These monotherapies typically target single pathways, neglecting the multifaceted nature of obesity, which involves dysregulation of appetite, energy expenditure, and fat metabolism [3,4]. The incomplete understanding of these complex interactions hinders the development of highly effective single-target therapies. A more holistic approach than current monotherapies is needed.

Combination therapies offer a more comprehensive strategy by targeting multiple pathophysiological mechanisms simultaneously. This approach aims to enhance weight loss, improve metabolic parameters, and increase the likelihood of sustained weight management. By addressing several contributing factors concurrently, combination therapies offer the potential to exceed the efficacy of monotherapies. The synergistic interactions between different agents can lead to a greater therapeutic benefit than the sum of their individual effects, improving patient adherence and long-term outcomes.

The remarkable success of bariatric surgery, such as Roux-en-Y gastric bypass (RYGB), in achieving significant and sustained weight loss, along with improvements in metabolic parameters, has stimulated extensive research into the underlying mechanisms [5]. While RYGB significantly alters gut hormone release, including a marked increase in postprandial GLP-1 levels, the observed metabolic benefits extend beyond the GLP-1 effect alone.

Studies have shown that GLP-1RA augmentation of RYGB or other duodenal exclusion procedures can lead to synergistic weight loss and metabolic improvements [6,7]. This synergy highlights the multifactorial nature of bariatric surgery’s success, involving complex interactions between various gut hormones and metabolic pathways. The altered nutrient absorption and gut hormone profiles induced by duodenal exclusion seem to complement the effects of GLP-1RAs, resulting in enhanced therapeutic outcomes.

GLY-200, an oral polymeric drug that targets the duodenal mucus barrier to emulate duodenal exclusion physiology was developed [8]. GLY-200’s mechanism of action is designed to mimic the metabolic benefits observed in bariatric surgeries and duodenal-jejunal bypass, providing a less invasive and more patient-friendly alternative. GLY-200 alters nutrient sensing and neurohormonal signaling through its barrier effect in the proximal small intestine. This affects incretin hormone secretion, potentially improving insulin sensitivity and glucose control. Changes in gut hormone release may also contribute to appetite suppression and reduced food intake.

A Phase 1 study in healthy volunteers showed that GLY-200 could mimic the biomarker signature observed after Roux-en-Y gastric bypass and duodenal exclusion devices [9]. A Phase 2a, randomized, double-blind, placebo-controlled, parallel-group study assessed GLY-200’s effects in 48 patients with T2D over 14 days. GLY-200 treatment resulted in significant reductions in fasting and postprandial blood glucose concentrations compared to placebo within those two weeks [10]. Improvements in fasting lipid profiles and weight loss were also noted. The Phase 2a results support GLY-200’s efficacy in improving key metabolic parameters in T2D patients and emulate duodenal exclusion physiology, making it a promising candidate for investigation, especially as a combination therapy.

In this article, the efficacy of GLY-200 and semaglutide, a GLP-1RA is evaluated in diet-induced obesity (DIO) mice, both as a monotherapy and in combination. Metabolic parameters including glycemia, body weight, body composition, food intake, and gut hormones are evaluated. The combination of GLY-200 with GLP-1RAs offers the potential to leverage the complementary mechanisms of these two distinct therapeutic approaches, leading to enhanced efficacy and potentially improved patient adherence and long-term outcomes.

## 2 Methods

### 2.1 Experimental design

The preclinical studies in DIO mice investigated GLY-200’s efficacy as a monotherapy and combination therapy with semaglutide (sema). Monotherapy and combination therapy experiments were conducted independently in different animal cohorts. Efficacy was determined by assessing changes in body weight (BW), fat mass (FM), lean mass (LM), food intake (FI), fasting glucose, and glycemic response to an oral glucose load (oGTT). All animal studies and experimental procedures were approved by the Institutional Animal Care and Use Committee (UM, PRO00011504).

### 2.2 Materials and reagents

GLY-200 was provided by Glyscend, Inc. (Lowell, MA). Semaglutide was from Bachem (Lot #: 3012999, Cat #: H-7894). All other materials were obtained from Sigma Aldrich or Fisher Scientific unless noted otherwise.

### 2.3 In vivo preclinical studies

Mice (C57BL/6J, Charles River) were singly housed in a standard 12/12 light/dark cycle.

In the first study (S1), DIO mice (age: 20wk, 60% kcal fat diet [D12492] for 14wk) received daily oral GLY-200 (QD, 20 mg/mouse, 200 μL, ∼425 mg/kg, *n* = 16, D1-28) or saline (QD, 200 μL, *n* = 10, D1-28) for 28 days after a 2-hr fast. From day 15–28, half of each group received subcutaneous semaglutide (Sema, 6 nmol/kg, SQ, QD, D15-28) in addition to their ongoing GLY-200 or saline treatment. Semaglutide was titrated as follows on D14, 2 nmol, on D15, 4 nmol, and on D16-28, 6 nmol. BW, FM, LM, FI, fasting glucose, and oGTT responses were assessed.

In the second study (S2), weight maintenance after semaglutide discontinuation was evaluated. DIO mice (age: 30wk, 60% kcal fat diet for 22wk) received subcutaneous semaglutide (sema, 6 nmol/kg, SQ, QD, *n* = 18, D1-15) from day 1–15. semaglutide was titrated as follows on D1, 1 nmol, on D2, 3 nmol, and on D3-14 or D3-28, 6 nmol. On day 15, the group was divided with mice receiving either daily oral GLY-200 (QD, 20 mg/mouse, 200 μL, ∼425 mg/kg, *n* = 10, D15-28), or saline (D15-28, SQ, *n* = 8). One group received both daily oral GLY-200 (QD, 20 mg/mouse, 200 μL, ∼425 mg/kg, *n* = 10, D1-28) and semaglutide treatment (sema, 6 nmol/kg, SQ, QD) from day 1–28. The control group received saline (*n* = 8, D1-28) from day 1–28 with the vehicle groups balanced on route of vehicle injection (PO vs SQ). All mice were dosed after a 2-hr fast. BW, FM, LM, FI, and fasting blood glucose and insulin were assessed at weeks 2 and 4.

### 2.4 Standardized OGTT protocol

A standardized OGTT protocol was utilized to evaluate efficacy. Prior to testing, mice were fasted 4–5 h, drug was given 1 h before oral glucose (2 g/kg). Blood was obtained from the tail vein, and blood glucose was measured with Biosen Glucose Analyzer (EKF Diagnostics). Blood glucose was collected at −60 (before drug), at 0 (before glucose), and at 15, 30, 60, and 120 min after glucose.

### 2.5 NMR

Fat and lean mass of all mice was analyzed using an EchoMRI Whole Body Composition Analyzer (Echo116Medical Systems, Houston, TX). Mice were scanned on day 1 (pre-dose), day 14, and day 28.

### 2.6 Fasting blood glucose and insulin

In Study 1, fasting blood and blood glucose were sampled after a 4–5 h fast at week 4.

In Study 2, mice were fasted 4–5 h to obtain blood glucose levels and insulin measures at weeks 2 & 4.

### 2.7 Statistical analysis

Statistical analysis was performed with the aid of GraphPad Prism (GraphPad Software). Unless noted, all values are expressed as Mean ± SEM, ∗∗∗∗p ≤ 0.0001, ∗∗∗p ≤ 0.001, ∗∗p ≤ 0.01, ∗p ≤ 0.05. For oral glucose tolerance tests, two-way ANOVA was applied to raw data to evaluate multiple comparisons between all groups and time points. AUC was calculated using the baseline glucose value as the zero Y parameter; percentage reduction was calculated manually. Data was analyzed using one way ANOVA, and the differences between means were analyzed using Tukey’s multiple comparisons.

## 3 Results

### 3.1 Monotherapy evaluation of oral duodenal exclusion therapy and GLP1R-Agonist treatment

**Figure 1:**
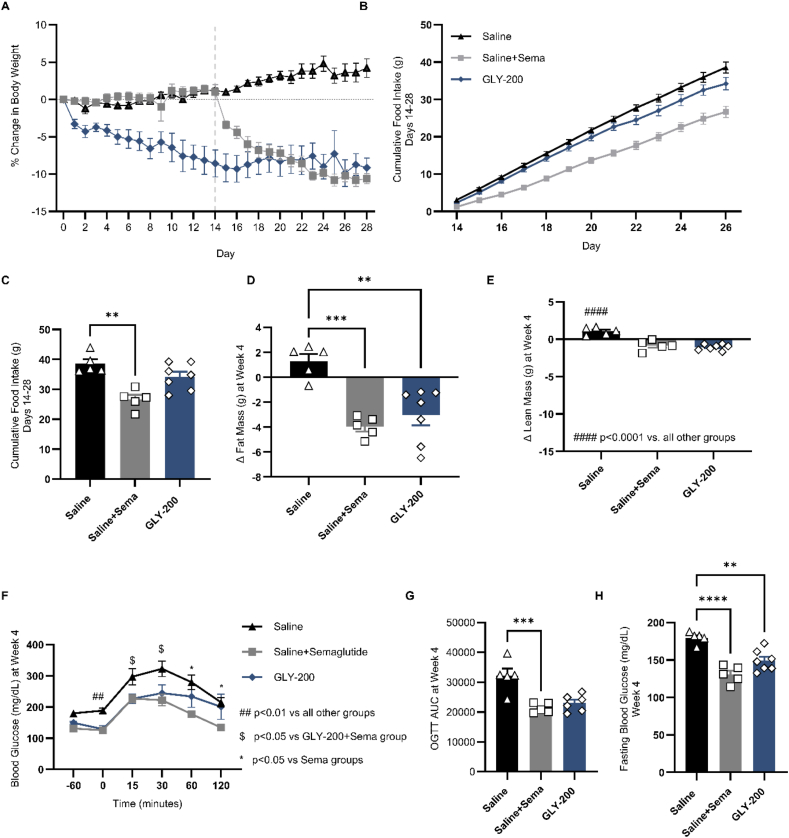
Impact of daily treatment with GLY-200 (20 mg/mouse, *N* = 8), semaglutide (sema, 6 nmol, SQ *N* = 8), or Saline (*N* = 10) as monotherapies on A) % change in body weight [gray dotted line at D14 indicates the start of semaglutide for the saline group], B–C) cumulative food intake (D14-28), D) delta fat mass, E) delta lean mass, F) oGTT, G) oGTT AUC, and H) fasting blood glucose at week 4. ∗*p* < 0.05, ∗∗*p* < 0.001, ∗∗∗*p* < 0.0001.

**Figure 2:**
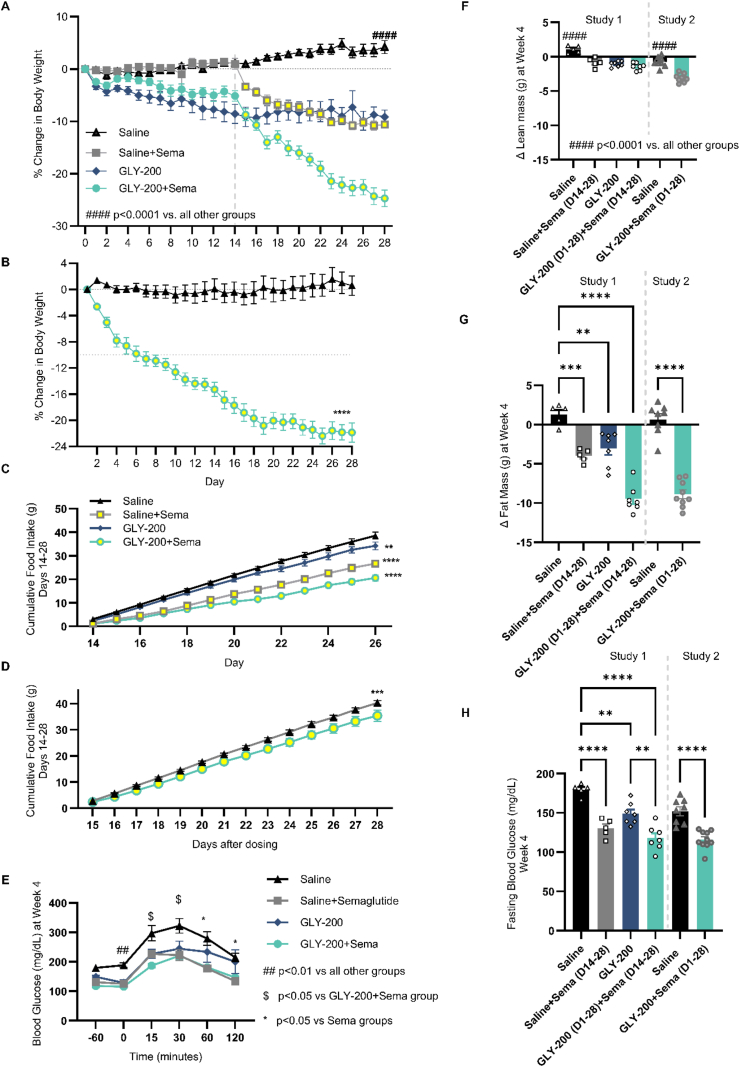
Impact of daily treatment GLY-200 (20 mg/mouse, *N* = 10) and semaglutide (sema, 6 nmol, SQ., *N* = 10) combination therapy or saline (*N* = 8) on A) % change in body weight S1, B) % change in body weight S2, C) cumulative food intake (D14-28) S1, D) cumulative food intake (D14-28) S2, E) oGTT, F) delta lean mass, G) delta fat mass, H) and fasting blood glucose at week 4. ∗*p* < 0.05, ∗∗*p* < 0.001, ∗∗∗*p* < 0.0001. Gray line delineates the addition of semaglutide to the group.

**Figure 3:**
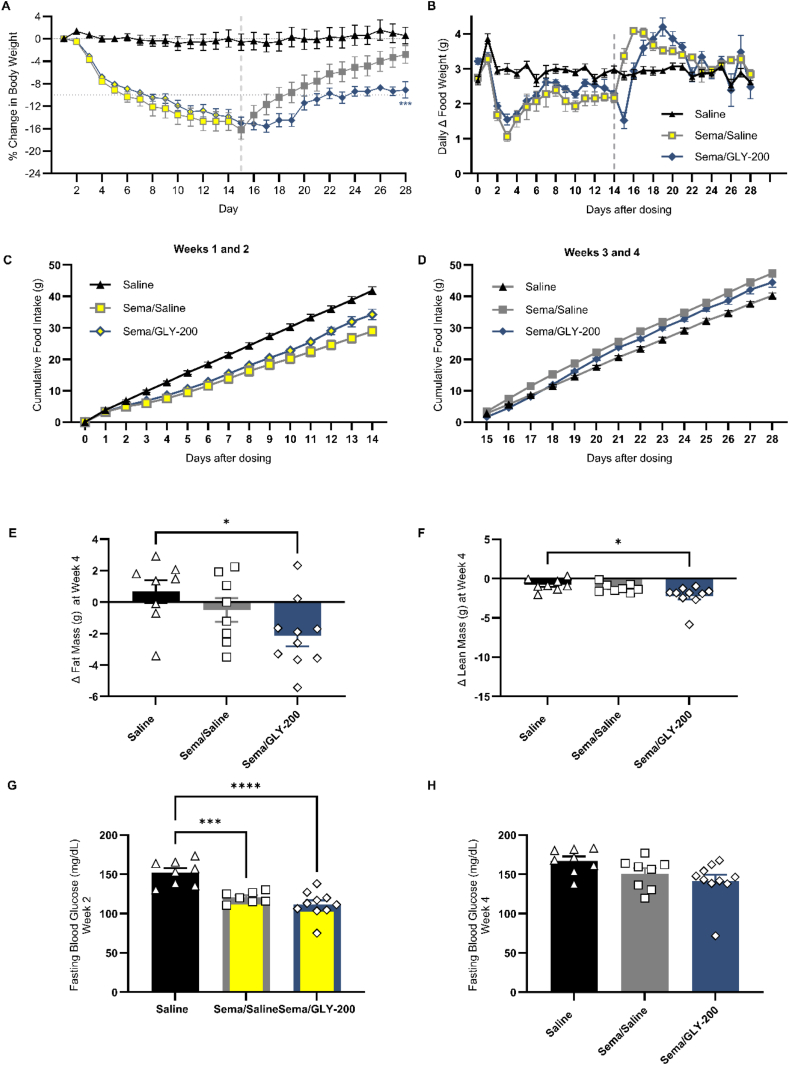
Impact of daily treatment of GLY-200 (20 mg/mouse) or saline on weight maintenance and glycemic control following sema discontinuation [day 15, delineated by gray dotted line]. A) % change in body weight, B) change in daily food intake, C) cumulative food intake D1-14, D) cumulative food intake D15-28, E) fat mass, F) lean mass, fasting blood glucose at G) week 2 and E, F, H) week 4. ∗*p* < 0.05, ∗∗*p* < 0.001, ∗∗∗*p* < 0.0001.

Monotherapy and combination therapy were evaluated in separate animal cohorts. Monotherapy experiment (Figure 1) offers a direct comparison of the two agents across multiple metabolic parameters, thereby setting the stage for the evaluation of combination therapy (Figure 2, Figure 3).

GLY-200 monotherapy significantly improved metabolic parameters in DIO mice without significantly reducing food intake (Figure 1). There was a slight decrease in FI with time, indicating GLY-200 may help reduce appetite with chronic use (Figure 1B). After day 28 of treatment, mice had reduced BW (↓13.4%) and FM (↓23.2%) with improved fasting blood glucose (↓17.0%) and incremental glucose AUC_0–120 min_ following oGTT (↓28.4%). Consistent with literature, treatment with Sema significantly decreased food intake (↓31.0%), BW (↓14.8%), and FM (↓28.8%) improving fasting blood glucose [11]. Both monotherapies were associated with a similar reduction in lean mass (GLY-200 ↓7.6%; sema ↓6.8%).

### 3.2 Combination therapy evaluation of oral duodenal exclusion therapy and GLP1R-Agonist treatment

The potential of combination therapy was investigated in two studies using different dosing paradigms (Study 1 and Study 2); the comparative results are presented here. The combination of GLY-200 and semaglutide produced even greater improvements compared to either treatment alone, indicating an additive effect. In Study 1 (Figure 2), semaglutide (sema: D15-28) was introduced into the dosing regimen after an initial 2 weeks on GLY-200 (GLY-200: D1-28). After 14 days, the combination had a significantly greater change in metabolic parameters than either monotherapy. Relative to semaglutide alone, the combination of semaglutide and GLY-200 significantly reduced BW, FM, and FI (↓14.1%, ↓28.6%, ↓33%, respectively) and significant improvement in fasting blood glucose. In Study 2 (Figure 2), GLY-200 and semaglutide were started simultaneously and elicited similar responses: reduced BW, FI and FM (↓22.4%, ↓12%, ↓47.9% respectively), with significantly improved FBG. Despite different dosing paradigms, both Study 1 and Study 2 yielded similar outcomes.

### 3.3 Weight maintenance via oral duodenal exclusion therapy following GLP1R-Agonist treatment discontinuation

To evaluate weight maintenance with GLY-200 after GLP1-RA treatment, semaglutide was administered for 14 days then discontinued and mice were switched to either saline or GLY-200 for an additional 14 days (Figure 3). In the initial 14-day treatment with sema, mice had lowered BW and FI. Following discontinuation of sema, food intake increased in both groups above the saline control and weight quickly rebounded in the group switched to saline, reaching similar BW and FM as the control group (Saline D1-28, BW 0.59%, FM 3.4% vs Sema/Saline BW -3.4%, FM -2.6%). In contrast, despite increased FI and an initial slight weight rebound in the GLY-200 group, after approximately 7 days, body weight plateaued and significant weight and FM reduction (BW -9.7%, FM -14.4%) was maintained compared to control. Both groups showed better responses to OGTT and FBG levels than the control group.

## 4 Discussion: clinical significance and future directions

The rationale for combination therapy in obesity management arises from the multifactorial nature of the disease. Obesity involves several overlapping pathophysiological processes that are unlikely to be fully corrected by a single agent. Combination approaches may therefore achieve greater efficacy by targeting complementary pathways, while allowing for lower doses of individual drugs and potentially fewer adverse effects [12,13]. In this context, pairing semaglutide, a centrally acting GLP-1 receptor agonist with GLY-200, an orally administered polymeric therapy designed to mimic duodenal exclusion physiology, offers a mechanistically distinct and synergistic strategy for achieving durable weight loss.

In the current study, we demonstrate that combining GLY-200 with semaglutide produces additive improvements in body weight, compared with either monotherapy alone. Notably, GLY-200 also helped maintain weight loss following semaglutide discontinuation, supporting its potential role as a maintenance therapy.

Clinically, GLP-1 receptor agonists have been shown to augment the weight-reducing and metabolic effects of bariatric surgery, including Roux-en-Y gastric bypass (RYGB) [6]. These additive and sometimes synergistic effects are thought to arise from the combination of independent mechanisms, central neurohormonal modulation by GLP-1RAs and peripheral metabolic reprogramming from duodenal exclusion [14]. Similarly, the complementary mechanisms of semaglutide and GLY-200 may recapitulate this dual-axis benefit through pharmacologic rather than surgical means.

While semaglutide primarily acts through central nervous system pathways to suppress appetite and improve glycemic control [15], GLY-200 likely exerts pleiotropic effects on the physiology of energy balance analogous to those observed after RYGB [16,17]. By forming a transient mucin-interacting barrier in the proximal small intestine, GLY-200 alters nutrient flow and intestinal signaling. This localized duodenal exclusion may modify gut hormone release, including endogenous GLP-1 and PYY, and enhance gut-brain communication pathways. These effects could potentiate the impact of exogenous GLP-1RAs such as semaglutide by increasing vagal or central GLP-1 receptor sensitivity [18,19].

Another complementary mechanism may involve improved pancreatic β-cell responsiveness to GLP-1 under duodenal exclusion physiology. Enhanced GLP-1 signaling in β-cells could strengthen insulin secretion and glucose tolerance while reducing hyperglycemia. Additionally, changes in nutrient sensing and enteroendocrine signaling may stimulate other anorexigenic gut hormones such as PYY, which further amplify the satiety response compared with semaglutide alone [[20], [21], [22]].

Together, these hypotheses suggest that the combination of GLY-200 with semaglutide acts through converging but independent pathways:

• **Neural:** potentiation of central GLP-1–mediated appetite suppression.
• **Peripheral metabolic:** enhancement of β-cell sensitivity and glucose control.
• **Gut-hormonal:** modulation of endogenous incretin and PYY release.
• **Physiologic remodeling:** induction of adaptive intestinal changes that sustain metabolic improvements.

Although these findings are promising, the studies were conducted in mice and over a relatively short duration. Translational limitations should be acknowledged, particularly regarding gastrointestinal tolerability, which cannot be fully assessed in animal models. Nonetheless, the consistent, large-magnitude effects across two independent experiments support the robustness of these results. These data provide a strong scientific rationale for human clinical trials to explore both combination and weight maintenance approaches.

## 5 Conclusion

The present findings highlight the potential of combining GLY-200, an oral pharmacologic duodenal-exclusion therapy, with GLP-1RA as a novel strategy for obesity and metabolic disease management. In diet-induced obesity models, the combination produced additive improvements in weight loss compared with either monotherapy. Importantly, GLY-200 also mitigated post-treatment weight regain following semaglutide withdrawal, suggesting a complementary role in sustaining long-term efficacy of incretin-based therapies.

Future clinical studies are warranted to validate these synergistic effects in humans and to define optimal dosing paradigms for co-administration. By targeting both central and intestinal pathways of metabolic regulation, the GLY-200 and GLP-1RA combination may overcome current limitations of single-agent approaches, providing a safe, durable, and patient-friendly treatment paradigm for obesity and type 2 diabetes.

## CRediT authorship contribution statement

**Taylor L. Carlson:** Writing – review & editing, Writing – original draft, Visualization, Validation, Resources, Methodology, Formal analysis, Conceptualization. **Mark Fineman:** Writing – review & editing, Visualization, Supervision, Project administration, Conceptualization. **Stace Kernodle:** Validation, Methodology, Formal analysis, Data curation. **Chelsea R. Hutch:** Validation, Resources, Methodology, Investigation, Formal analysis, Data curation. **Christine Bryant:** Writing – review & editing, Visualization, Conceptualization. **Kevin Colbert:** Conceptualization. **Randy J. Seeley:** Writing – review & editing, Visualization, Supervision, Project administration, Methodology, Investigation, Formal analysis, Conceptualization. **Ashish Nimgaonkar:** Writing – review & editing, Writing – original draft, Supervision, Project administration, Methodology, Funding acquisition, Conceptualization.

## Funding

This work was supported by Glyscend Therapeutics.

## Declaration of competing interest

The authors declare the following financial interests/personal relationships which may be considered as potential competing interests: TLC, MF, CB, KC, AN report equity or stock options in Glyscend. RJS has received research support from Novo Nordisk, Fractyl, AstraZeneca, Congruence Therapeutics, Eli Lilly, Bullfrog AI, Glyscend Therapeutics, Diasome and Amgen. RJS has been a paid consultant for Novo Nordisk, Eli Lilly, CinRx, Fractyl, Structure Therapeutics, Crinetics, Amgen, Congruence Therapeutics, Heliocore, Gallant and Nuanced Health. RJS has equity in Nuanced Health, Coro Bio, Eccogene, Fractyl and Rewind. The research was funded by Glyscend Therapeutics. There are no patents or intellectual property to report.

## Data Availability

The data that has been used is confidential.

## References

[1] Zheng Z., Zong Y., Ma Y., Tian Y., Pang Y., Zhang C. (2024). Glucagon-like peptide-1 receptor: mechanisms and advances in therapy. Signal Transduct Targeted Ther.

[2] Yao H., Zhang A., Li D., Wu Y., Wang C.-Z., Wan J.-Y. (2024). Comparative effectiveness of GLP-1 receptor agonists on glycaemic control, body weight, and lipid profile for type 2 diabetes: systematic review and network meta-analysis. BMJ.

[3] Verdam F.J., Greve J.W.M., Roosta S., Eijk H. van., Bouvy N., Buurman W.A. (2011). Small intestinal alterations in severely Obese hyperglycemic subjects. J Clin Endocrinol Metabol.

[4] Heymsfield S.B., Wadden T.A. (2017). Mechanisms, pathophysiology, and management of obesity. N Engl J Med.

[5] Cefalu W.T., Rubino F., Cummings D.E. (2016). Metabolic surgery for type 2 diabetes: changing the landscape of diabetes care. Diabetes Care.

[6] Pratama K.G., Nugroho H., Hengky A., Tandry M., Pauliana P. (2024). Glucagon-like peptide-1 receptor agonists for post-bariatric surgery weight regain and insufficient weight loss: a systematic review. Obes Med.

[7] Jirapinyo P., Jaroenlapnopparat A., Zucker S.D., Thompson C.C. (2024). Combination therapy of endoscopic gastric remodeling with GLP-1RA for the treatment of MASLD. Obes Surg.

[8] Carlson T.L., Colbert K., Vieira M., Guerina F.V., Bryant C.L.N., Habegger K. (2025). Development of a targeted oral pharmacologic duodenal exclusion therapy for the treatment of metabolic diseases. Sci Adv.

[9] Fineman M.S., Bryant C.L.N., Colbert K., Jozefiak T.H., Petersen J.S., Horowitz M. (2023). First-in-human study of a pharmacological duodenal exclusion therapy shows reduced postprandial glucose and insulin and increased bile acid and gut hormone concentrations. Diabetes Obes Metabol.

[10] Bryant C.L.N., Colbert K., Hompesch M., Chaves S., Nimgaonkar A., Fineman M.S. (2025). GLY-200, an oral pharmacologic duodenal exclusion drug, resulted in positive effects on glucose, lipids and bodyweight in patients with type 2 diabetes: results of a randomized, double-blind placebo-controlled trial. Diabetes Obes Metabol.

[11] Gabery S., Salinas C.G., Paulsen S.J., Ahnfelt-Rønne J., Alanentalo T., Baquero A.F. (2020). Semaglutide lowers body weight in rodents via distributed neural pathways. JCI Insight.

[12] Camilleri M., Acosta A. (2018). Combination therapies for obesity. Metab Syndr Relat Disord.

[13] Melson E., Miras A.D., Papamargaritis D. (2023). Future therapies for obesity. Clin Med.

[14] Gimeno R.E., Briere D.A., Seeley R.J. (2020). Leveraging the gut to treat metabolic disease. Cell Metab.

[15] Sisley S., Gutierrez-Aguilar R., Scott M., D’Alessio D.A., Sandoval D.A., Seeley R.J. (2014). Neuronal GLP1R mediates liraglutide’s anorectic but not glucose-lowering effect. J Clin Investig.

[16] Albaugh V.L., He Y., Münzberg H., Morrison C.D., Yu S., Berthoud H.-R. (2023). Regulation of body weight: lessons learned from bariatric surgery. Mol Metabol.

[17] Mosinski J.D., Kirwan J.P. (2016). Longer-term physiological and metabolic effects of gastric bypass surgery. Curr Diabetes Rep.

[18] Wachsmuth H.R., Weninger S.N., Duca F.A. (2022). Role of the gut–brain axis in energy and glucose metabolism. Exp Mol Med.

[19] Krieger J.-P. (2020). Intestinal glucagon-like peptide-1 effects on food intake: physiological relevance and emerging mechanisms. Peptides.

[20] Habegger K.M., Al-Massadi O., Heppner K.M., Myronovych A., Holland J., Berger J. (2014). Duodenal nutrient exclusion improves metabolic syndrome and stimulates villus hyperplasia. Gut.

[21] Muñoz R., Carmody J.S., Stylopoulos N., Davis P., Kaplan L.M. (2012). Isolated duodenal exclusion increases energy expenditure and improves glucose homeostasis in diet-induced obese rats. Am J Physiol Regulat Integr Compar Physiol.

[22] Laferrère B., Pattou F. (2018). Weight-independent mechanisms of glucose control after Roux-en-Y gastric bypass. Front Endocrinol.

